# Effects of focused ultrasound pulse duration on stimulating cortical and subcortical motor circuits in awake sheep

**DOI:** 10.1371/journal.pone.0278865

**Published:** 2022-12-13

**Authors:** Hyun-Chul Kim, Wonhye Lee, Kavin Kowsari, Daniel S. Weisholtz, Seung-Schik Yoo

**Affiliations:** 1 Department of Radiology, Brigham and Women’s Hospital, Harvard Medical School, Boston, MA, United States of America; 2 Department of Artificial Intelligence, Kyungpook National University, Daegu, South Korea; 3 Department of Mechanical Engineering, Massachusetts Institute of Technology, Cambridge, MA, United States of America; 4 Department of Neurology, Brigham and Women’s Hospital, Harvard Medical School, Boston, MA, United States of America; University of Colorado Anschutz Medical Campus, UNITED STATES

## Abstract

Low-intensity transcranial focused ultrasound (tFUS) offers new functional neuromodulation opportunities, enabling stimulation of cortical as well as deep brain areas with high spatial resolution. Brain stimulation of awake sheep, in the absence of the confounding effects of anesthesia on brain function, provides translational insight into potential human applications with safety information supplemented by histological analyses. We examined the effects of tFUS pulsing parameters, particularly regarding pulse durations (PDs), on stimulating the cortical motor area (M1) and its thalamic projection in unanesthetized, awake sheep (*n* = 8). A wearable tFUS headgear, custom-made for individual sheep, enabled experiments to be conducted without using anesthesia. FUS stimuli, each 200 ms long, were delivered to the M1 and the thalamus using three different PDs (0.5, 1, and 2 ms) with the pulse repetition frequency (PRF) adjusted to maintain a 70% duty cycle at a derated *in situ* spatial-peak temporal-average intensity (I_spta_) of 3.6 W/cm^2^. Efferent electromyography (EMG) responses to stimulation were quantified from both hind limbs. Group-averaged EMG responses from each of the hind limbs across the experimental conditions revealed selective responses from the hind limb contralateral to sonication. The use of 0.5 and 1 ms PDs generated higher EMG signal amplitudes compared to those obtained using a 2 ms PD. Faster efferent response was also observed from thalamic stimulation than that from stimulating the M1. Post-sonication behavioral observation and histological assessment performed 24 h and 1 month after sonication were not indicative of any abnormalities. The results suggest the presence of pulsing scheme-dependent effects of tFUS on brain stimulation and attest its safety in awake large animals.

## Introduction

Low-intensity transcranial focused ultrasound (tFUS) has emerged as a promising non-invasive brain stimulation technique that modulates excitability of region-specific neural tissue with excellent spatial selectivity (on the order of millimeters) and with the ability to reach deep brain areas [1–3]. The utility and safety of the technique have been established in small [4–7] and large animal models [8–10], as well as in non-human primates [11, 12]. Recently, an increasing number of studies on healthy human volunteers have been reported [13–17] with transition to potential clinical applications, such as amelioration of disorders of consciousness [18] and epilepsy [19].

Given at an acoustic intensity below the level that may generate heat, tFUS is typically applied in packets of short-duration pulses to achieve neuromodulation [14, 17, 20–23] whereby acoustic waves having a specific pulse duration (PD) are applied repeatedly at a pulse repetition frequency (PRF). Pulse repetitions have shown superior stimulation efficiency over continuous sonication (*i*.*e*., 100% duty cycle) [7, 24] while the use of PDs in a range of a few milliseconds (0.3–5 ms) at 50–70% duty cycle (*i*.*e*., the proportion of active sonication time given per stimulation), given in durations of 200–500 ms, has shown to modulate region-specific brain excitability [7, 9, 20, 25]. Although the detailed mechanism and effects of the ultrasonic neuromodulation await further examination, studies have revealed that sonication parameters such as fundamental frequency and pulsing schemes influence the modulatory efficiency [7, 23, 24, 26]. For example, acoustic intensity thresholds [7, 24], calcium signal responses [23], and synaptic transmission [26] have been identified to be dependent on the fundamental frequency of ultrasound. In terms of pulsing scheme, a study on *ex vivo* mouse brain slices revealed that the pulsed sonication administered with a 0.4 ms PD at 60% duty cycle (corresponding PRF of 1,500 Hz) yielded higher neuronal response rates than those from using 2 ms PD given at the same duty cycle [23].

Efforts are being made to better understand the effects of pulsing scheme on stimulation efficacy of tFUS. FUS has been shown to selectively stimulate regional brain activity in large animal models such as sheep [27] and pigs [8]; however, the effects of pulsing parameters, particularly the choice of PD on the stimulation efficiency, warrant further investigation. As the responses to FUS stimulation in rodents have been reported to be dependent on the type and the depth of anesthesia [5, 25], experiments in the awake state are desired to reduce the anesthetic confounders in neuronal activity [28, 29].

The present study examines the effects of different PDs (0.5, 1, and 2 ms) on stimulating the primary motor cortical area (M1) and the corresponding thalamic circuit in fully awake sheep. A wearable, custom-fit FUS transducer headgear [27] was used to deliver acoustic stimulation to the targeted brain regions over multiple sonication sessions. The electromyography (EMG) amplitudes and response rates resulting from the FUS stimulations were measured, and histological evaluation of the brain tissues, together with post-sonication behavioral monitoring, were conducted to evaluate the safety of the method.

## Materials and methods

### Animals

The study was carried out in compliance with ethical guidelines set forth by the Institutional Animal Care and Use Committee of Brigham and Women’s Hospital (Protocol #: 2020N000113). Only female sheep (*n* = 8, Polypay, New England Ovis, LLC, NH, 20.7 ± 4.6 weeks, weight = 39.1 ± 3.9 kg, mean ± standard deviation—s.d.) were used because males may grow scurs that can excessively interrupt/absorb sonication. Sheep were housed in a vivarium under 12:12h light:dark cycles without restrictions on food/water intake except for those necessitated by the magnetic resonance imaging (MRI) acquisition needed for image-guidance of sonication.

### Wearable FUS transducer and neuroimage-guided FUS targeting

**Fig 1:**
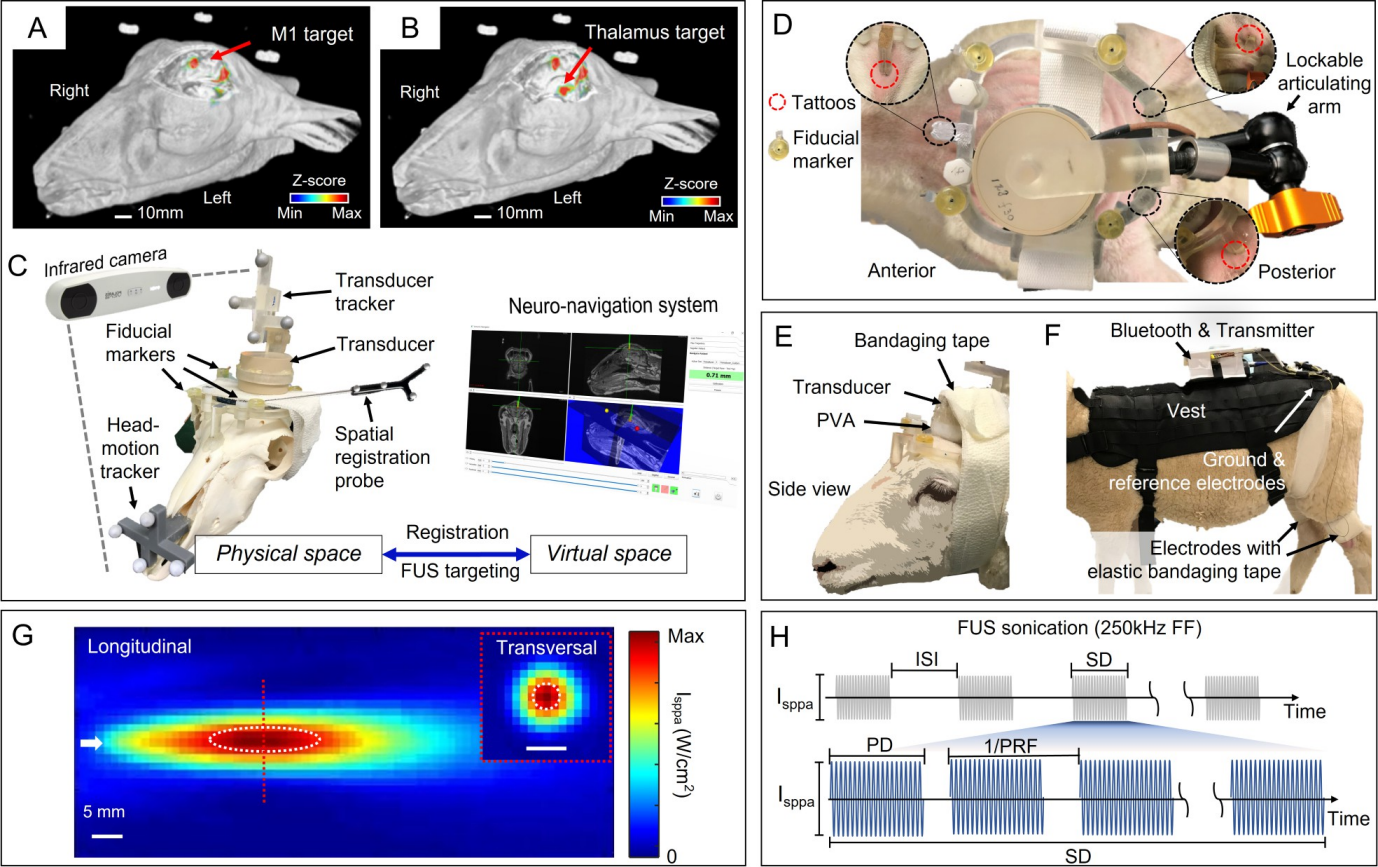
Schematics of the experimental settings with neuro-navigation. Examples of functional maps overlaid on 3D anatomical neuroimaging data, showing the locations of the (A) M1 and (B) thalamic areas (z-score > 2.58; *p* < 0.01), as indicated by the red arrows. (C) Illustration of the neuro-navigation system. (D) Top view of the wearable FUS headgear worn by the sheep. Three tattoo markings on the scalp, used for headgear positioning, shown with the corresponding support bases of the headgear. (E) Side view of sheep wearing the FUS headgear secured by bandaging tape. (F) Experimental setup for EMG acquisition. A vest was used to attach a telemetry EMG Bluetooth transmitter and preamplifier. The ground and reference electrodes were placed on the back, and an electrode was placed over the gastrocnemius of each hind limb. The electrodes were secured using elastic tape. (G) Spatial profiles of the acoustic intensity across the longitudinal and transversal planes. The region bound by 90%-maximum of the intensity is depicted with the dashed white line. The arrow indicates the direction of sonication. (H) Illustration of acoustic parameters: fundamental frequency (FF), inter-stimulation interval (ISI), spatial-peak pulse-average intensity (I_sppa_), sonication duration (SD), pulse duration (PD), pulse repetition frequency (PRF).

A single-element FUS transducer (GPS200-MR, Ultran Group, State College, PA) operating at a fundamental frequency of 250 kHz was used to generate ultrasound. The detailed method of operation and characterization of the acoustic field are described elsewhere [20]. Briefly, a pulsed electrical sinusoidal waveform was generated by two serially connected function generators (33500B, Keysight, Santa Rosa, CA) to actuate the transducer after being amplified by a linear power amplifier (240L, Electronics and Innovations, Rochester, NY). The signal was transmitted to the transducer *via* an impedance-matching box (JT-800, Electronics and Innovations). The acoustic intensity profile of the transducer was characterized in a degassed water tank covering the longitudinal (30 × 70 mm^2^, 1 mm step size) and transversal (30 × 30 mm^2^, 1 mm step size) planes along the sonication path, using a needle-type hydrophone (HNC200, Onda, Sunnyvale, CA) mounted on a 3-axis motorized robotic stage. The ellipsoidal acoustic focus, 3 mm in diameter and 13 mm in length (defined at the area bound by 90%-maximum intensity), was located 30 mm away from the exit plane of the transducer surface (Fig 1G). A compressible cone-shaped polyvinyl alcohol (PVA) hydrogel (two freeze-thaw cycles, 9% weight per volume in degassed water, 341584, MilliporeSigma, St. Louis, MO) was made in-house, and its thickness was adjusted to fill the gap between the transducer and the scalp for adequate acoustic coupling.

The M1 and thalamus of the left hemisphere were the sonication targets under the image-guidance. Detailed methods are described elsewhere [27]. First, the scalp surface, after shaving, was marked with three tattoo points (one on the snout, mid-line rostral to the eyes, and two on the skin medial to the ears) to facilitate reproducible positioning of the 3D-printed headgear that contained four MR fiducial markers (PinPoint®, Beekley Crop., Bristol, CT) for image registration and guidance (Fig 1D). The headgear was custom-made to fit the animals’ individual head anatomies, and high-resolution head MRI (3 Tesla MRI, Skyra, Siemens, Munich, Germany) was then performed to image the head of sheep wearing their custom-fit headgear secured by elastic bandaging tape. Each sheep-specific location of target areas in the brain was identified based on anatomical and functional MRI following the method previously described [9, 10]. The functional activation map and the structural neuroanatomy were used to guide the placement of the acoustic focus to the desired brain areas using a sheep skull model (Fig 1A–1C) [27]. The spatial error of the co-registration between the real and virtual spaces from neuro-navigation, represented as fiducial registration error (FRE), was 0.51 ± 0.11 mm (mean ± s.d.) across the animals (*n* = 8).

### FUS sonication

Brain stimulation was performed over multiple sessions (5 sessions per animal) across a period of 7.5 ± 2.7 days (*n* = 8 sheep; mean ± s.d.). We used 70% DC and a 200 ms sonication duration (SD) per stimulation trial. In each session, four different stimulation parameters, having three PDs (0.5, 1, and 2 ms), including no-sonication (labeled ‘no-FUS’ herein), were given with 4.8 s intervals (randomized and balanced among trial types). 50 stimulation trials per parameter and per session were given while the sequence of stimulation, including no-FUS trials, was computer-generated, and experimenters were blinded to the stimulation type. Thus, the total number of stimulation trials per parameter was 250. The acoustic intensity, given with sufficient inter-stimulus intervals (≥ 4.8s), has not been shown to generate any thermal effects on neither the brain tissue nor the skull [9]. No anesthesia was used. Prior to sonication, the sheep were acclimated for 8.5 ± 6.0 days to wearing the headgear with a mock-up transducer (Fig 1E) as well as a fabric harness vest (TG-526, OneTigris, Shenzhen, China). The vest was needed to guide the animal and to attach a telemetry EMG device (gMOBIlab+, g.tec, neurotechnology, Albany, NY; Fig 1F). Wool covering the hind limbs around the gastrocnemii muscle, lower back, and scalp were sheared and shaved for EMG electrode placement and sonication. The EMG device was attached to the vest, and a surface cup electrode (g.LADYbird, g.tec, neurotechnology) was placed on the shaved area of each hind limb while the ground and reference electrodes were located on their lower back, coupled with conductive gel (g,GAMMAgel, g.tec, neurotechnology). All electrodes were wrapped and secured during sonication experiments with elastic bandages (Fig 1F). The headgear was then placed on the scalp aligned with the tattoo markings and secured also with elastic bandages. Ultrasound gel (Aquasonic, Parker Laboratories, Fairfield, NJ) was applied between all interfaces (*i*.*e*., the PVA coupler and scalp/transducer surfaces).

During the experiments, animals stayed still with an experimenter gently holding the harness vest. Three different types of stimulations, each 200-ms in duration, were applied using three different PDs (0.5, 1, and 2 ms) at *in situ* spatial-peak pulse-average intensity (I_sppa_) of 5.2 W/cm^2^ (corresponding spatial-peak temporal-average intensity, I_spta_, of 3.6 W/cm^2^). The *in situ* I_sppa_ was estimated using an intensity transmission level of 25.6% derived from ovine skull transmission at the same fundamental frequency [9]. A schematic diagram of the sonication pulsing parameters is illustrated in Fig 1H. The 70% duty cycle was selected as it generated the highest stimulation efficacy among sheep regardless of their anesthetic status [9, 27]. To maintain the 70% duty cycle for each stimulation, the PRF was adjusted to 1,400, 700, and 350 Hz for 0.5, 1, and 2-ms PDs, respectively.

### Processing and analysis of EMG data

EMG data from awake large animals without physical constraints, particularly in the context of FUS-mediated neuromodulation, inevitably contain erroneous confounders such as inadvertent motion-related signals unrelated to the applied FUS stimulation. We adopted a data analysis technique to select the FUS trials and corresponding EMG signal that respond to the stimulation [27]. In brief, raw EMG data acquired at a sampling rate of 256 Hz were filtered by 20-Hz high-pass and 60-Hz notch filters to reduce the confounders, such as cardiorespiratory and power line noise [30]. Full-wave rectification and temporal Gaussian smoothing with a 44-ms window were then applied [5]. Based on our previous study [9], the processed EMG data were segmented in a time-locked fashion covering 200 ms before and 800 ms after the onset of sonication, and were screened for the presence of (1) signal saturation (*i*.*e*., absolute value ≥ 100 μV), (2) a single peak with short latencies (< 25 ms, which is related to the possible presence of reflex-type startle), and (3) multiple peaks or synchronous EMG signals from both hind limbs (postural motion-related artifacts that are unrelated to sonication) within a time-window of 25–250 ms after the FUS onset. The screened EMG data from each trial were baseline-corrected with respect to the signal mean obtained from the pre-FUS segment (covering -200 ms to 0 ms of the sonication onset).

The baseline-corrected EMGs from both hind limbs were subsequently selected based on the following inclusion criteria: (1) |amplitude| ≤ 5σ during the pre-FUS segment and (2) amplitude ≥ 4σ in the FUS segment [27]. The included EMG features were time-averaged per animal. The EMG data obtained under the no-FUS condition were averaged without response selection. The standard deviation, σ, was derived from a Gaussian distribution fitted to the EMG amplitude distribution across the pre-FUS segments of the EMG data and used as a baseline signal during the resting state [27]. We note that (1) the selected data were, therefore, not necessarily synchronized with the sonication and (2) negative signal amplitudes, although rectified during the data preparation, indicate reduced EMG activity compared to that acquired during the pre-FUS segment. The ‘response rate’ per PD condition was defined as the number of trials that showed stimulation-related EMG responses (regardless of the side of the hind limbs) with respect to the number of stimulatory trials without showing the signal saturation. We further constructed a histogram of stimulation-related EMG onset latency from each animal. The EMG onset latency was defined at the time when EMG exceeded the resting-state σ after the sonication onset. To evaluate the time-wise differential effect of PDs, one-way analysis of variance (ANOVA) followed by Tukey-Kramer *post-hoc* analysis was conducted on each time point of EMG amplitudes across the experimental conditions. Significance was defined with *p* < 10^−3^ after Bonferroni correction.

## Results

### EMG processing and derivation of response events

**Fig 2:**
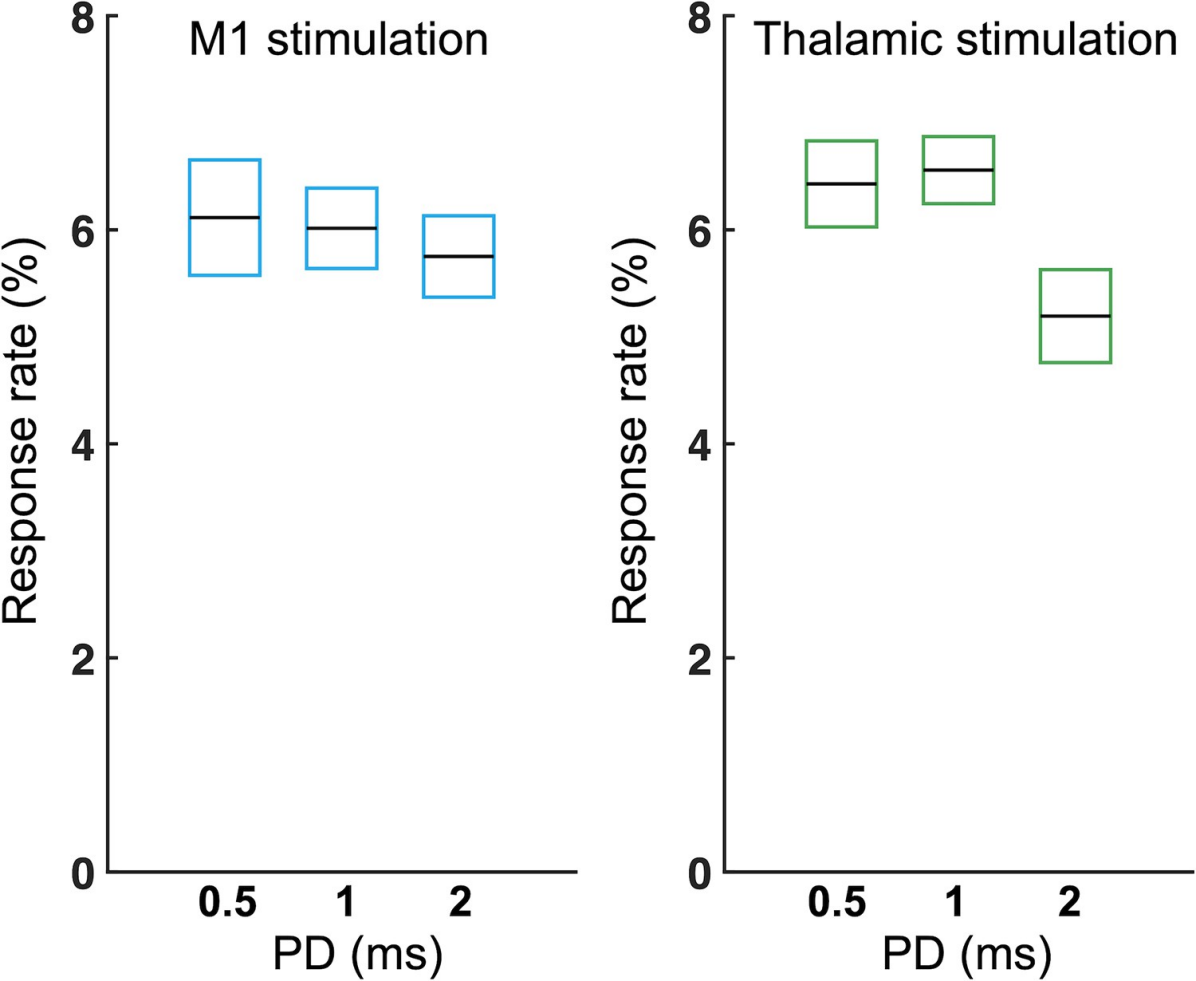
Group-averaged EMG response rates from FUS stimulations of the M1 and thalamic areas, comparing the effects of various PDs. Horizontal black lines and boxes indicate averaged response rate and standard errors, respectively (*n* = 8). Significant differences were not found in the response rates across the experimental conditions (one-way ANOVA, *p* > 0.05).

We did not detect any apparent limb movements that synchronized with sonication across the animals; however, sonication-related EMG features were observed from all animals. The EMG-based group-averaged response rate is shown in Fig 2 (with detailed information in Table 1). The total number of EMG data sets after excluding motion-related artifacts was 221.5 ± 7.9 and 224.7 ± 7.5 (out of 250; mean ± s.d.; *n* = 8) for the M1 and thalamic stimulations, respectively. M1 stimulation yielded averaged response rates of 6.1 ± 1.5%, 6.0 ± 1.1%, and 5.8 ± 1.1%, from 0.5, 1, 2 ms PDs, respectively. There was no statistical response rate difference across the PD conditions (one-way ANOVA, *p* = 0.81). The averaged response rates resulting from thalamic stimulations were 6.4 ± 1.1% (0.5 ms PD), 6.6 ± 0.9% (1 ms PD), 5.2 ± 1.2% (2 ms PD). Similar to the results obtained from the M1 stimulation, statistical differences in the response rates to thalamic stimulation were not found across the PD conditions (one-way ANOVA, *p* = 0.07).

**Table 1:** Summary of response events, total number of data sets, and response rates (in percentage) measured from the M1 and thalamic stimulations.

	M1 (Response event/Total data set*/Response rate)			Thalamus (Response event/Total data set*/Response rate)		
ID	0.5 ms PD	1 ms PD	2 ms PD	0.5 ms PD	1 ms PD	2 ms PD
1	20/226/8.8	16/226/7.1	13/230/5.6	14/235/6.0	17/239/7.1	11/236/4.7
2	11/224/4.9	15/227/6.6	14/219/6.4	12/225/5.3	13/223/5.8	15/226/6.6
3	13/215/6.0	10/200/5.5	12/200/6.0	18/208/8.7	17/216/7.9	11/204/5.4
4	10/222/4.5	13/213/6.1	16/215/7.4	13/224/5.8	14/227/6.2	8/220/3.6
5	14/224/6.3	15/219/6.8	14/222/6.3	13/228/5.7	17/219/7.7	12/221/5.4
6	18/229/7.9	15/228/6.6	13/230/5.7	16/222/7.2	14/229/6.1	10/232/4.3
7	11/222/5.0	13/218/6.0	10/222/4.5	16/224/7.1	13/224/5.8	10/234/4.3
8	13/235/5.5	9/229/3.9	9/222/4.1	13/231/5.6	13/224/5.8	16/222/7.2
Mean	13.8/224.6/6.1	13.4/220.0/6.0	12.6/220.0/5.8	14.4/224.7/6.4	14.8/225.1/6.6	11.6/224.4/5.2
*s*.*d*.	3.5/5.8/1.5	2.4/9.9/1.1	2.3/9.5/1.1	2.1/8.0/1.1	1.9/7.0/0.9	2.7/10.3/1.2

*The data sets were selected based on screening for the presence of signal saturation (*i*.*e*., absolute value ≥ 100 μV) from a total of 250 FUS stimulations. PD: pulse duration, s.d., standard deviation.

### EMG amplitude and onset latency distribution analysis

**Fig 3:**
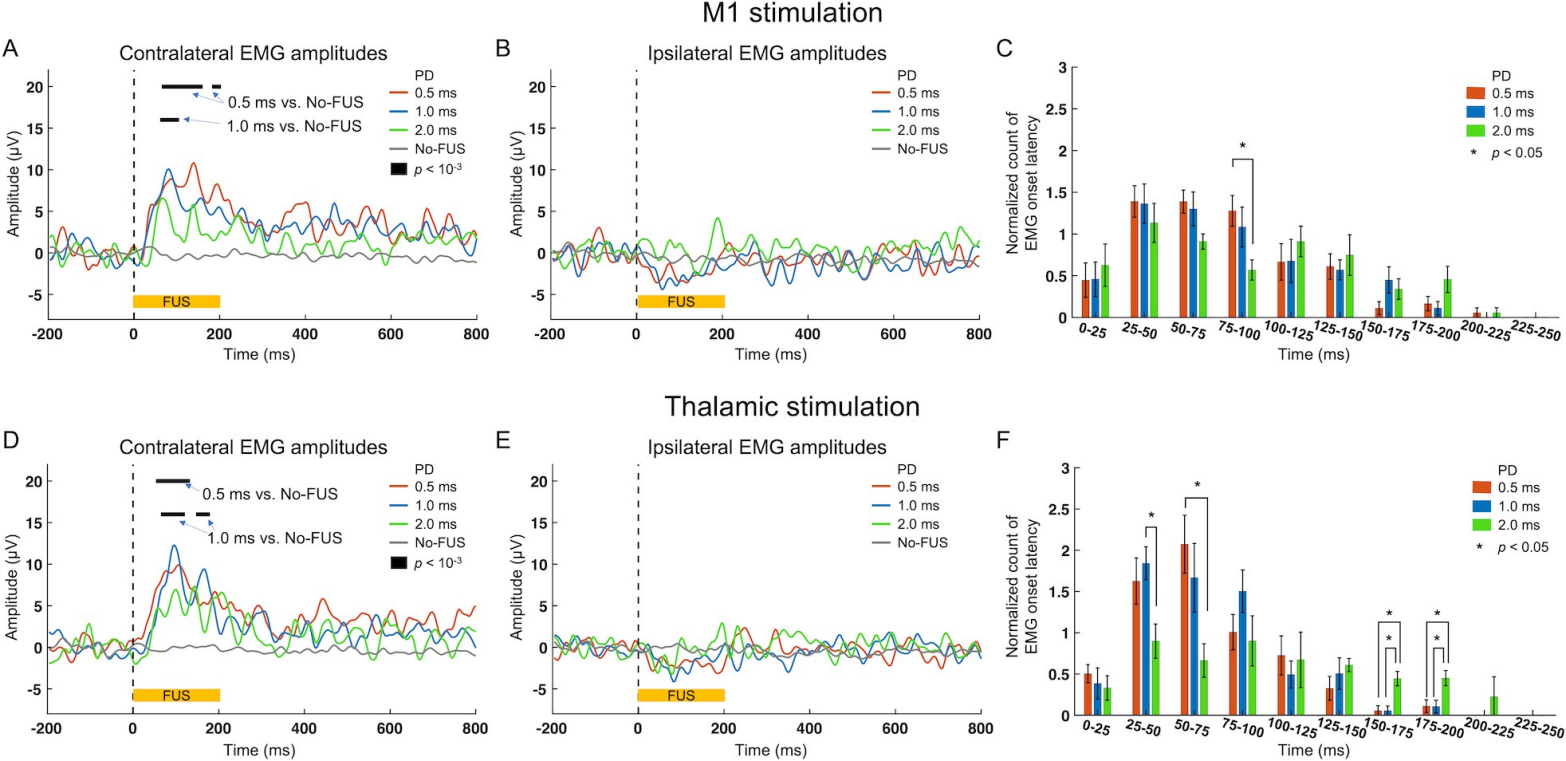
Time-locked EMG amplitudes and averaged onset latency distributions of EMG response. (A, B, C, E) Group-averaged EMG amplitudes across the experimental conditions using pulse durations (PDs) of 0.5 ms (red lines), 1 ms (blue lines), and 2 ms (green lines) and the no-FUS condition (black lines), from the hind limbs (A, D) contralateral and (B, E) ipsilateral to sonication to the M1 and thalamic areas, respectively. Horizontal black bars indicate regions of significant differences in the EMG amplitudes between the experimental conditions (*p* < 10^−3^, Tukey-Kramer *post-hoc* analysis following one-way ANOVA). (C, F) Group-averaged (*n* = 8), normalized histogram of EMG onset latency measured from the right hind limb (contralateral to the M1 sonication). *p < 0.05, Tukey-Kramer *post-hoc* analysis following one-way ANOVA.

Group-averaged amplitudes of EMG responses to the stimulation of the M1, acquired from both hind limbs, are shown in Fig 3A across the PD conditions. Examination of EMG amplitude from the right hind limb (contralateral to sonication) revealed that the 0.5-ms PD condition generated higher EMG signal amplitudes than those obtained from the no-FUS condition in the time segments of 69.5–155.5 ms and 186.7–198.4 ms. Sonication using 1-ms PD yielded significantly higher EMG amplitudes compared to those from the no-FUS condition in the time segments of 65.6–100.8 ms. Evaluation of EMG acquired from the left hind limb (ipsilateral to sonication), on the other hand, showed no statistical differences across all conditions (Fig 3B). Although statistically not significant, we noted a slight reduction in the EMG amplitude from the hind limb ipsilateral to sonication, which was qualitatively observable during sonication using 0.5- and 1-ms PDs. The histogram of stimulation-related EMG onset latency across the PD conditions (Fig 3C), normalized to total number of screened EMG for each animal, showed that most of EMG onsets were observed over the time segments of 25–100 ms. In particular, the PD of 0.5 ms yielded more frequent responses having 75–100 ms onset latency than those from the PD of 2 ms (one-way ANOVA *p* < 0.05, followed by Tukey-Kramer *post-hoc* analysis *p* < 0.05) (Fig 3C).

An identical analysis was conducted to examine group-averaged EMG responses to thalamic stimulation. Sonication given using a PD of 0.5 ms significantly increased the group-averaged EMG amplitudes from the right hind limb compared to those of the no-FUS condition in the time segment of 57.8–128.1 ms. The 1-ms PD condition generated significantly increased EMG signals compared to those from the no-FUS condition in the time segments of 69.5–116.4 ms and 151.6–175.0 ms (Fig 3D). On the other hand, from the left hind limb (ipsilateral to sonication), no statistical differences among the PD conditions were found (Fig 3E). Similar to the M1 stimulation, we also noted a slight reduction in the EMG amplitude (not statistically significant) from the hind limb ipsilateral to SD the use of 0.5 and 1 ms PDs. In terms of latency distribution of EMG onsets, thalamic stimulation generated more frequent onsets at 25–75 ms bins compared to the other time bins. In the examination of the onset latency distribution across the PD conditions (Fig 3F), we found that the use of 1 ms PD resulted in more frequent EMG onsets in the 25–50 ms segment while the use of 0.5 ms PD also yielded more frequent onsets in the 50–75 ms segments than those from the 2 ms PD (one-way ANOVA *p* < 0.05, followed by Tukey-Kramer *post-hoc* analysis, all *p* < 0.05). Mainly being attributed to the nearly absent responses from 0.5 and 1 ms PD conditions, we found that the 2 ms PD condition showed more frequent response onset latency in 150–200 ms segments.

### Comparison of onset latency distribution between M1 and thalamic stimulations

**Fig 4:**
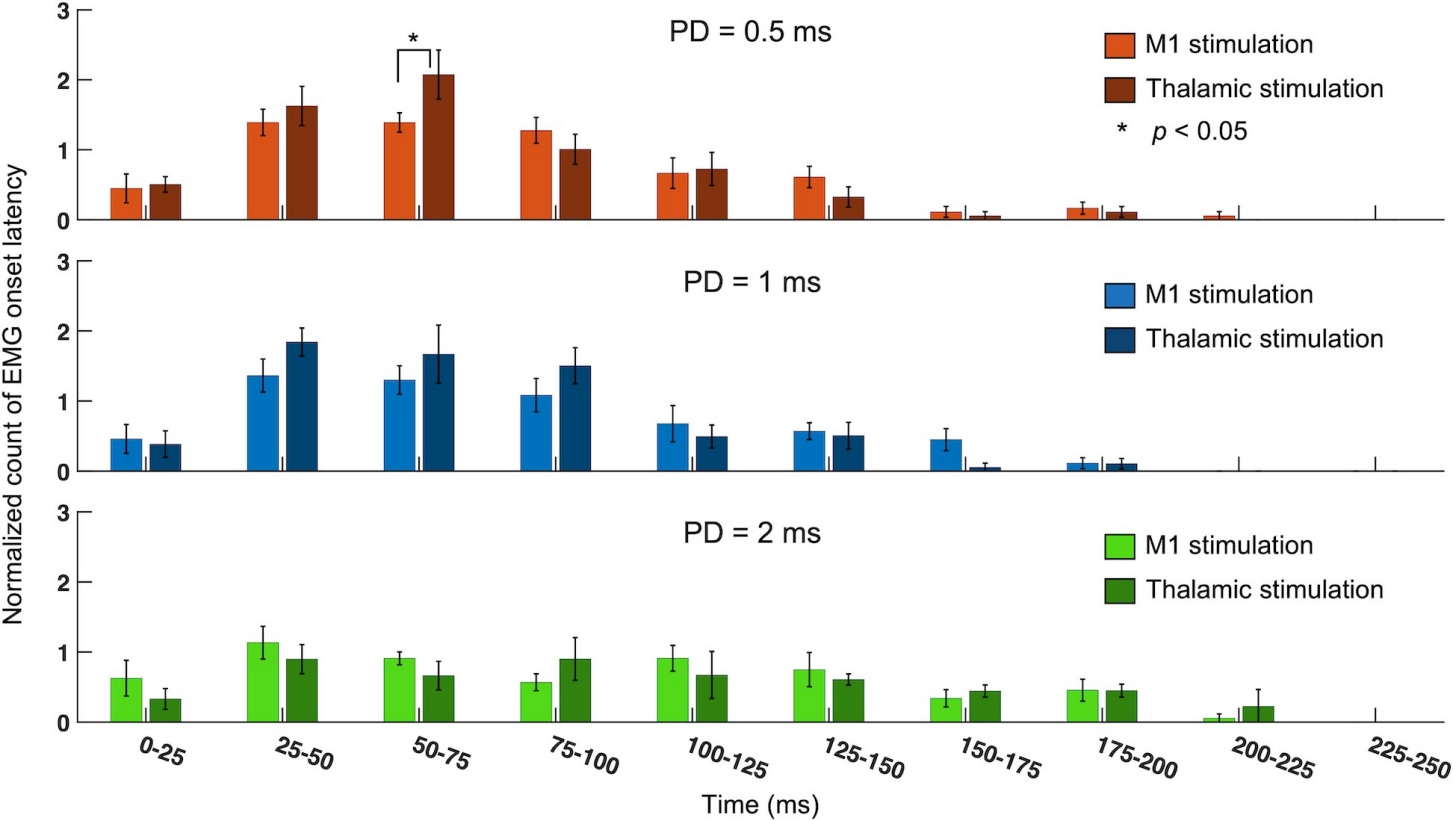
Comparison of the onset latency distributions between M1 and thalamic stimulation. Group-averaged (*n* = 8), normalized histogram of EMG onset latency measured from the right hind limb (contralateral to the M1 sonication). *p < 0.05, one-tailed two-sample *t*-test.

The comparison of the EMG onset latency distribution between M1 and thalamic stimulation (Fig 4) revealed that the use of 0.5-ms PD for the thalamic stimulation yielded more frequent response onsets than those of the M1 stimulation in 50–75 ms segment (*p* < 0.05; one-tailed two-sample *t*-test). However, there were no significant differences between M1 and thalamic stimulation among other time segments.

## Discussion

The present study investigated the effects of PD on acoustic stimulation of motor circuits, both M1 and thalamus, among awake sheep in eliciting EMG responses. The sheep brain model offers excellent translational potential in acoustic brain stimulation due to their relatively large brain volume, cranial and neuroanatomical similarities with those of humans [31, 32], as well as the availability of various neuropathological models [33–35].

### Effects of pulse duration on EMG response rates

The response rates elicited by M1 and thalamic stimulations (within a range of 5.8–6.1% and 5.2–6.4%, respectively) were comparable to EMG response rates reported in previous studies involving awake [27] and anesthetized sheep [9]. We did not, however, find any differential effects from the PD on the response rate itself. This result was different from the previous study on anesthetized sheep that showed higher response rates to stimulation of the somatosensory brain circuits through the use of 0.5 ms PD (at 70% duty cycle) compared to the use of longer PDs (1, 2, and 3 ms) [9]. We conjecture that elimination of anesthetic confounders in the present study, may have attributed to these more uniform response rates; however, the differences in study design (*i*.*e*., acoustic intensities as well as stimulated areas of the brain) might have also contributed to our current findings.

### Effects of pulse duration on EMG amplitudes

We observed that the M1 stimulations using 0.5 ms or 1 ms PDs significantly increased EMG amplitudes from the hind limb contralateral to FUS stimulation in the range of 65.6–198.4 ms segment (Fig 3A). This indicates faster EMG responses to FUS among awake sheep compared to that observed in anesthetized sheep (~100 ms; conducted under identical pulsing parameters [9]). The use of 2 ms PD, despite a slightly higher EMG amplitude compared to the no-FUS condition, did not generate differential EMG responses. As the EMG amplitude is related to the number of recruited neuromuscular units [36], it serves as important indicator for brain stimulation efficiency. Thus, our findings suggest the existence of parameter-dependent efficiency in acoustic brain stimulation, whereby the use of a 0.5-ms PD given at 1,400 Hz PRF (*i*.*e*., 70% duty cycle) or a 1-ms PD given at 700 Hz PRF yields superior motor unit recruitment compared to a longer PD (2 ms; corresponding PRF = 350 Hz) at the same duty cycle. These findings are congruent with observations from a previous study on excised mouse brain slices (transgenic mice to express fluorescence sensitive to calcium signaling) that showed pulsed sonication with a 0.4-ms PD (at 60% duty cycle) generated higher neuronal response rates than those of a 2-ms PD [23]. Another *in vivo* study on rats reported that pulsed sonication utilizing a 0.5-ms PD elicited motor responses at the lowest acoustic intensity compared to the stimulation given at different (shorter or longer) PDs at the same duty cycle (70%) [7], which agreed well with the existence of PD-dependent stimulation efficiency.

The observed increases in the EMG amplitude from the left hind limb (contralateral to M1 sonication) reflect the selective efferent response to unilateral tFUS brain stimulation. Although statistically not significant, we noted a slight reduction in the EMG amplitude from the hind limb ipsilateral to sonication (using 0.5- and 1-ms PDs). Interestingly, we found similar phenomena in our previous study that involved a different group of sheep (Fig 4 in [27]). As to this consistent reduction of EMG amplitude from the hind limb ipsilateral to sonication, we hypothesize that stimulation of the unilateral motor areas may reduce the activity of contralateral circuits (*via* inhibition) through extensive anatomical/functional interhemispheric connections [37, 38]. Further interrogation of potential interhemispheric inhibitory effects by FUS is needed to verify this postulation. FUS stimulation of the thalamus yielded similar results to the M1 stimulation, which suggested the existence of specific PDs (*e*.*g*., 0.5 or 1 ms PD) to attain a higher stimulation efficiency. Studies on non-human primates and humans that examine the pulsing-scheme dependent stimulation efficiency would help characterize the similarities and differences in response to acoustic brain stimulation across the different species.

### Effects of pulse duration on EMG onset latency distributions

Assessment of the EMG onset distributions (Fig 3C and 3F) revealed prevalent onset latency observed in the time segments of 25–100 ms, consistent with the results from previous studies among awake sheep [27]. Generation of stimulation-related EMG amplitudes in shorter onset latencies observed in awake animals, compared to those measured from anesthetized sheep [9], also support our conjecture on faster recruitment of larger-diameter muscle fibers (thus generating higher EMG amplitudes) and faster efferent responses to FUS stimulations in awake state [39].

Based on the comparisons of EMG onset latency among different PD conditions, thalamic stimulation yielded differential onset latency in earlier time segments (25–75 ms) than the M1 stimulation (Fig 3C and 3F). When onset latency counts were compared between M1 and thalamic stimulations (Fig 4), more frequent EMG onsets were seen from thalamic stimulation in the 50–75 ms segment when using 0.5 ms PD. This faster EMG onset may reflect a shorter neural conduction distance from the thalamus to the hind limb (compared to that from the M1). However, contributions from different excitatory mechanisms (and neuronal signal conduction time) between the cortex and the thalamus cannot be excluded as a potential cause [40]. Comparative evaluation of the acoustic stimulation of *ex vivo* cortical/thalamic tissue, along with further studies in cell-type specific neural responses would benefit the elucidation of the causes for the observation.

### Post-sonication monitoring and histological assessment

**Fig 5:**
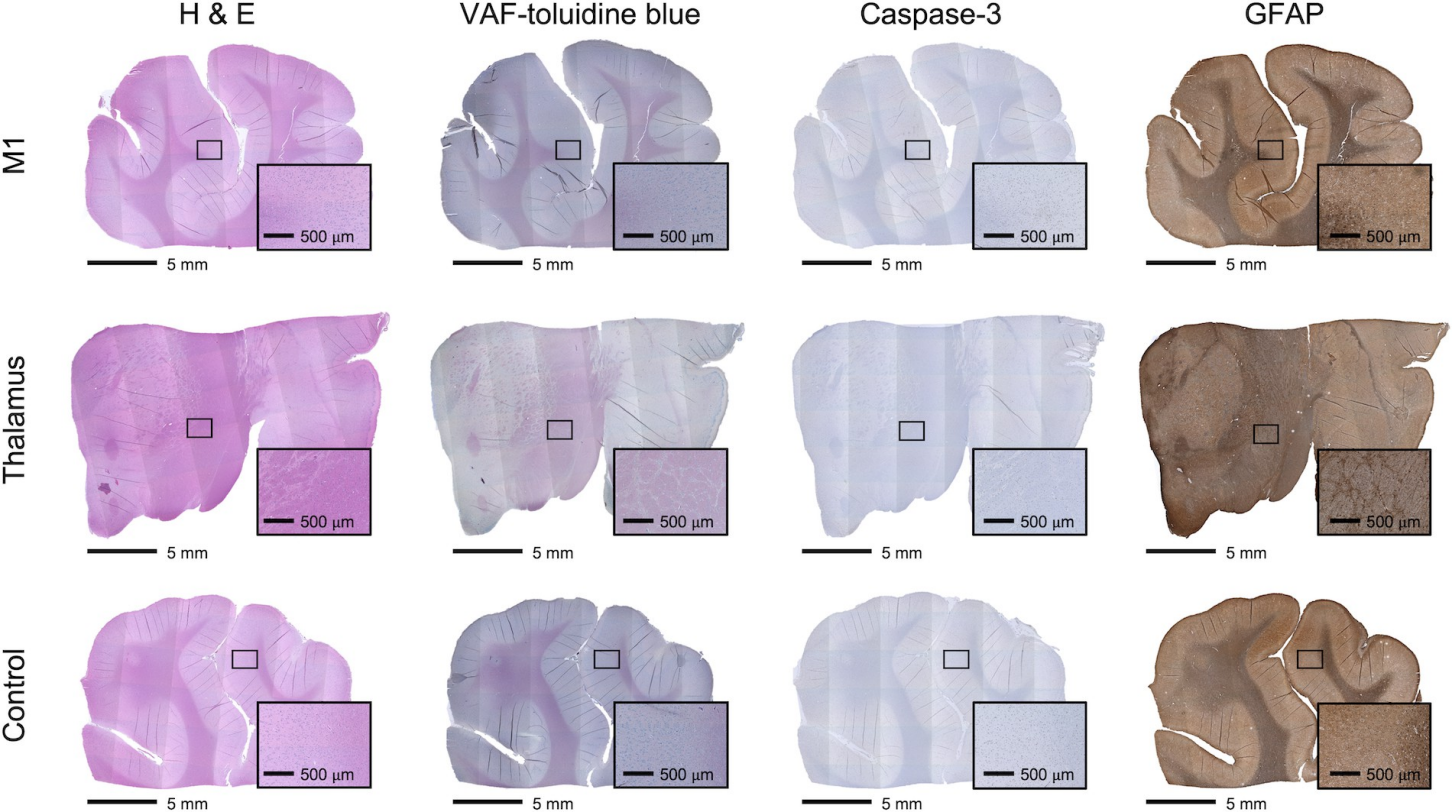
Exemplar histology results of the brain tissue (‘ID#1’; sacrificed one month after the last FUS session). Mosaic H&E, VAF-toluidine blue, caspase-3 and GFAP (from left to right column) stained microscopic images of the M1 (top row), thalamus (middle row) and control area (bottom row) are shown. Inset: magnified image from a region-of-interest (a rectangular area).

The *in situ* I_sppa_ of 5.2 W/cm^2^ (corresponding *in situ* I_spta_ of 3.6 W/cm^2^) used in the present study would not raise temperature of the sonicated skull and the brain, based on the numerical simulation of thermal effects [9]. The sheep were euthanized at different time intervals (*n* = 4 within 24 h, and *n* = 4 for 1 month) after the last sonication session. During their survival period, behavioral and body conditions were monitored once every 1–3 days. None of the animals showed abnormal behavior (*e*.*g*., chewing slats or chains, lethargy, uninterested in feeding, restlessness) throughout the study period. Euthanasia was performed using an intravenous injection of sodium pentobarbital solution (0.22 mL/kg) under anesthesia with an intramuscular injection of Telazol/xylazine. Following tissue harvest and sectioning, we conducted histology analysis using hematoxylin and eosin (H&E; to assess tissue necrosis/hemorrhaging), vanadium acid fuchsin (VAF)-toluidine blue staining (to detect ischemic response), and immunohistochemistry (IHC) of glial fibrillary acidic protein (GFAP; to detect glial cell infiltration after tissue damage) and caspase-3 (to detect apoptotic activity), according to the procedure described previously [9]. The histological assessments indicated no sign of brain tissue damage (Fig 5). This is consistent with a recent study on the histologic safety of FUS neuromodulation using sheep (*in situ* I_spta_ of 0.6–13.8 W/cm^2^ with 50% or 100%, 550 kHz, ExAblate 2100) [22] and primates (*in situ* I_spta_ of 0.4–25.8 W/cm^2^ at 50% duty cycle, 270 kHz single-element) [22]. The results also suggest that the multiple FUS brain stimulation sessions (7.5 ± 2.7 days; 5 sessions per animal) can be safely conducted in awake sheep under the present sonication conditions.

### Limitations of the study

Several limitations of the study, which shared similar features with our initial work on awake sheep [27], are noteworthy, and listed herein—(1) potential misalignment of the acoustic focus from the target due to unintended headgear movement, (2) elongated acoustic focal shape that may lead to stimulation of additional brain regions, especially during thalamic stimulation, and (3) the lack of individual derivation of derating factors for estimating acoustic intensity through numerical simulation. Adoption of implanted FUS transducers or a phase-array transducer configuration would be advantageous in reducing some of these experimental confounders to enhance the accuracy and reproducibility in the placement of acoustic focus. Elaborate numerical simulation of acoustic propagation through skull [31], accompanied by the acquisition of cranial information using computed tomography (CT) or ultra-short TE (UTE) MRI [41, 42], can be used to estimate the animal-specific *in situ* acoustic intensity and its location.

We did not find that the stimulation was accompanied by any movements of the hind limbs. The *in situ* I_sppa_ of 5.2 W/cm^2^ (corresponding *in situ* I_spta_ of 3.6 W/cm^2^) used in the present study was comparable or even lower than those used in previous studies that did not elicit visible responses from anesthetized (I_sppa_ of 3–18.2 W/cm^2^ [9, 10]) or awake sheep (I_sppa_ of 20.5 W/cm^2^ [27]). We speculate that the number of motor units in the brain recruited by sonication was insufficient to cause any visible muscle responses. As the magnitude of EMG responses is positively correlated with acoustic intensities [10], we hypothesize that the use of higher acoustic intensity may eventually yield visible muscle activity. However, caution is advised when adopting higher acoustic intensities, which may induce undesired heat-related or mechanical damage to the sonicated neuronal tissues.

Although the sequence of the brain stimulation parameter, given at 4.8 s intervals was randomized and balanced across multiple sessions, the stimulation could be exerting its downstream effects through long-term potentiation or long-term depression of action potentials [43, 44]. Furthermore, the stimulation using different PDs may confound the outcome of subsequent stimulations. For instance, Yu and colleagues reported the types of neurons having differential sensitivities to sonication parameters [45], which suggests the use of uniform sonication parameters within each session would reduce the potential confounders in response measures. Thus, future work would benefit from using a fixed set of sonication parameters per session (with sufficient time intervals between sessions) and subsequent evaluation of intra- and inter-session reproducibility of responses to the stimulation.

There is growing evidence that the FUS delivery can produce auditory confounds [46, 47] that need to be included in the experimental design. This is a critical aspect as EMG responses could be produced by external factors (such as environmental equipment noise that could change when FUS is delivered). Although single-peak EMG responses having short latencies < 25 ms were excluded from present analysis to reduce the contribution from possible presence of reflex-type startle [48], further investigation on the effect of auditory phenomena in acoustic brain stimulation should be considered in the future studies.

## Conclusions

We investigated excitatory effects of FUS on the M1 and its thalamic projection in awake sheep by quantifying changes in EMG amplitudes with varying PDs. The safety of multiple FUS sessions was demonstrated through comprehensive histological analyses, which provided useful information on the technology’s translational potential. To our knowledge, this is the first study to demonstrate the differential excitatory efficacy of FUS with respect to the use of different PDs in a large animal model without confounding factors from anesthesia. The use of different PDs operating at the same duty cycle may impose differential mechanical impact on mechanosensitive ion channels as well as on membrane capacitance (and, thus capacitive displacement currents) [49, 50]. For instance, differential mechanosensitive neural calcium responses toward ultrasound pulse duration were observed [51] while the inward-rectifier potassium current activation and inactivation of neuronal cells were found to be dependent on acoustic intensity [52]. Further investigation to link the dependency of neuronal activation on pulsing schemes is warranted to better-understand the underlying mechanism of our observation. As neuromodulatory FUS also possesses the capability to suppress regional brain activity, further studies on examining the effects of different pulsing parameters on suppression are desired. As the neuromodulatory capability of FUS may incite a new mode of neurotherapeutics for various neurological and psychiatric disorders, probing the effects of its pulsing parameters in humans is also coveted.
